# Human Posture Estimation: A Systematic Review on Force-Based Methods—Analyzing the Differences in Required Expertise and Result Benefits for Their Utilization

**DOI:** 10.3390/s23218997

**Published:** 2023-11-06

**Authors:** Sebastian Helmstetter, Sven Matthiesen

**Affiliations:** Karlsruhe Institute of Technology (KIT), IPEK—Institute of Product Engineering, 76131 Karlsruhe, Germany; sebastian.helmstetter@kit.edu

**Keywords:** human pose prediction, activity recognition, motion capture, classification, machine learning, digital human model, virtual sensor, biomechanics, pressure sensor

## Abstract

Force-based human posture estimation (FPE) provides a valuable alternative when camera-based human motion capturing is impractical. It offers new opportunities for sensor integration in smart products for patient monitoring, ergonomic optimization and sports science. Due to the interdisciplinary research on the topic, an overview of existing methods and the required expertise for their utilization is lacking. This paper presents a systematic review by the PRISMA 2020 review process. In total, 82 studies are selected (59 machine learning (ML)-based and 23 digital human model (DHM)-based posture estimation methods). The ML-based methods use input data from hardware sensors—mostly pressure mapping sensors—and trained ML models for estimating human posture. The ML-based human posture estimation algorithms mostly reach an accuracy above 90%. DHMs, which represent the structure and kinematics of the human body, adjust posture to minimize physical stress. The required expert knowledge for the utilization of these methods and their resulting benefits are analyzed and discussed. DHM-based methods have shown their general applicability without the need for application-specific training but require expertise in human physiology. ML-based methods can be used with less domain-specific expertise, but an application-specific training of these models is necessary.

## 1. Introduction

Human posture estimation is an essential method for data acquisition in many applications, including the fields of sports science, ergonomics, rehabilitation and user-centered product design [1,2,3,4,5,6]. Traditionally, camera-based motion capture systems have been used for human motion capturing and posture estimation [7,8]. Camera-based systems, especially marker-based systems, offer high precision but are limited by the need for external measurement equipment such as cameras, leading to a restricted measurement space and significant preparation effort [9,10]. However, in recent years, non-camera-based sensors have also emerged as valuable alternatives for human posture estimation [11,12,13]. Non-camera-based sensors can offer advantages in terms of flexibility and portability as well as cost-efficiency [14,15,16].

Common alternatives to camera-based methods include the use of data from inertial measurement units (IMUs), electromyography (EMG), and pressure or force sensors [17,18,19]. The sensors for measuring these data do not need to be set up externally to observe the subject. They can be worn directly on the human body or be integrated into smart products like smartwatches, phones, or even sensor-integrated devices such as exoskeletons or smart seats [17,20,21,22,23]. While estimating human posture using IMU sensors for dynamic motion has been well investigated and is used in some commercial motion-capturing systems for sports analyses and the film industry, this technology has limitations when capturing static postures [20,24]. For static postures, such as lying in a bed, sitting on a chair or standing in the same position for a long period of time while working, the displacement of the measurement data is small. Thus, the drift of the sensors has a large impact on the accuracy of the posture estimation [25].

New approaches investigate the application of force and pressure data from pressure and force sensors or as a virtual force vector in simulations to estimate human posture in static applications. Since the first approaches were published in the early 2000s through the dissemination of machine learning algorithms (ML) and digital human models (DHM), research in this area is relatively new. As a result, many models and methods for force-based human posture estimation has emerged in various research disciplines. However, there is currently a lack of a comprehensive overview of these methods, as well as criteria for selecting the most suitable models and methods for specific applications. Such an overview is necessary to fully exploit the potential of this technology and to guide further research in this area. To address this gap, this systematic literature review aims to summarize the existing models and methods in the literature and answer the following research questions:

1. What are the existing input data sources and estimation methods used in studies to estimate human posture based on force data and pressure maps?
2. Which human posture estimation methods are used for which types of application scenarios, and what previous expert knowledge is required for their use?

## 2. Human Posture Estimation

**Figure 1:**
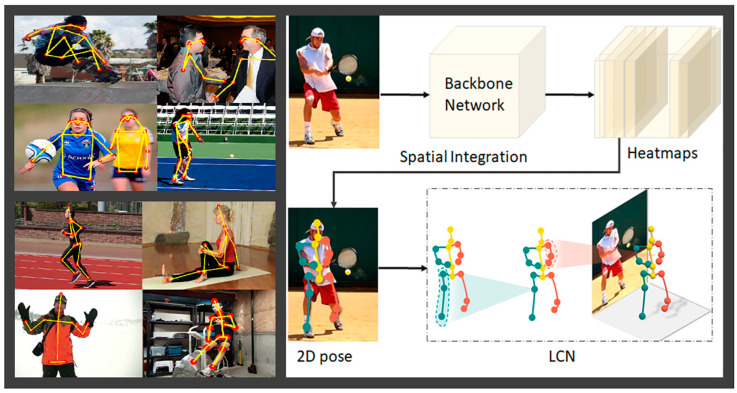
A general schemata for the data processing of human posture estimation in computer vision on 2D rgb images (**right hand side**) and examples of identified human postures in diverse activity contexts (**left hand side**) described in a graphical abstract by Ben Gamra and Akhloufi [26] (Reprinted with permission from [26], 2023 Elsevier).

Human posture estimation, the process of determining the spatial configuration of the human body, has diverse applications across various domains, e.g., computer vision, sports analyses, biomechanical research, security, and surveillance. It involves identifying the positions and orientations of body parts, typically using visual or sensor data. The term “human posture estimation” or “human pose estimation” is most commonly known by camara-based computer vision applications [26,27], as shown in Figure 1. In computer vision, it is crucial for tasks like gesture recognition and human–computer interaction by the identification of the human joint positions or body shapes from rgb or depth images [28]. However, human posture estimation is not limited to cameras, as described so far.

**Figure 2:**
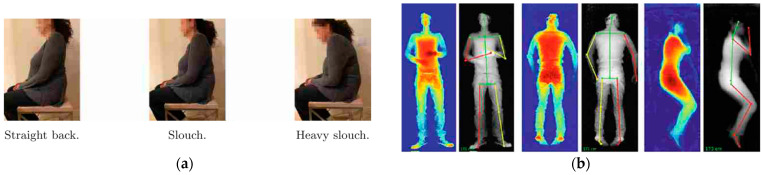
Different human postures that are estimated based on force data in representative studies in the literature review. (**a**) Human sitting postures classified by pressure data from a sensor on top of a chair [29]; (**b**) Sleeping postures that can estimated using ML-based algorithms [13] (Reprinted with permission from [13,29], 2023 Springer Nature).

In the following literature review, the term “human posture estimation” is generally used for refer to the detection of human posture (joint angles, sitting positions, sleeping positions, etc.) from a measured data set. Examples of slouch detection and sleeping posture are shown in Figure 2. In the case of our literature review, the data on which the estimation is based are limited to pressure maps and force vectors. We also included studies from the representative literature for the review that use activity recognition and re-infer the most probable posture. This method can be applied if the activity is dominated by instinctive movements like gait activities and is not influenced by external boundary conditions, like in the case of a working posture. It is unique for a certain task and working environment.

## 3. Materials and Methods

**Figure 3:**
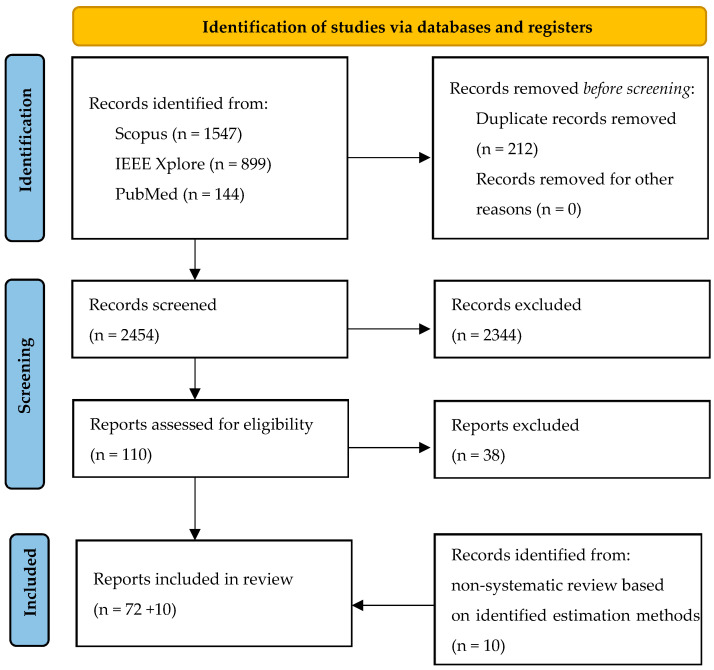
Flowchart of the review process according to PRISMA 2020 [30].

This systematic review follows the criteria of the “Preferred Reporting Items for Systematic Reviews and Meta-Analyses (PRISMA 2020)” statement [30,31]. The steps of the review process according to PRISMA 2020 are illustrated in Figure 3.

### 3.1. Information Sources and Searching Strategy

This systematic literature review is performed on publications between 2000 and 2022 using the databases of Scopus, IEEE Xplore and PubMed. A versatile string is used for the review due to differences in the declaration of the methods. Combining synonyms of the five major terms “human”, “posture”, “estimation”, “force”, and “method” (Table 1) leads to the following string: TITLE-ABS-KEY (human AND (posture OR pose) AND (prediction OR estimation OR classification OR recognition) AND (force* OR pressure OR load OR weight) AND (model* OR method*)). To limit the results to non-camera-based methods and exclude studies on human blood pressure, the following keywords are used: “*rgb*”, “*video*”, “*vision*”, “*cular”, “*blood*” and “*birth*”. Further, the results are limited to the subareas “*medicine*”, “*engineering*”, “*computer science*”, “*health professions*”, “*multidisciplinary*” and “*nursing*”. Other subareas are checked by random samples and excluded due to their lack of relevance. The language is limited to English.

### 3.2. Screening Process

Searching in both databases resulted in a total of 1365 papers. To select the relevant ones, the papers are summarized by title, author and abstract in a spreadsheet using EXCEL 2019 (Microsoft Corporation, Redmond, WA, USA). The following exclusion criteria are defined for screening the primary results by title and abstract:

- The minimum required input for posture estimation includes data from at least one force sensor, pressure mat or virtual force vector.
- The output of the estimation includes the human posture, e.g., whole-body postures or one or more joint angles.
- Additional sensor inputs are allowed, except human motion data from classical motion capture systems. Classical motion capture systems are only allowed for the evaluation of the results.

Non-relevant papers are excluded from further steps. After selecting the relevant papers by title and abstract, the full text reports of the remaining papers are manually screened again during the data analysis. No automated tools are used for the screening process. The literature is managed using the software CITAVI 6 (Swiss Academic Software GmbH, Wädenswil, Switzerland). As a result, a total of 72 papers, each representing a distinct study, are selected for further data analysis.

### 3.3. Additional Reports with Non-Standardized Terminology

Due to the use of inconsistent terminology in describing ML-based and DHM-based methods, 10 additional papers on force-based human posture estimation are included after the screening process. This non-systematic literature review is based on the main methods, models and applications that are identified by analyzing the results of the structured literature review. A valuable input for additional relevant papers is provided by a systematic literature review by Ngueleu et al. [32]. This review also covers some methods of force-based human posture estimation from insole pressure data, but the main focus in this review is on insole force sensors and methods for step-counting. Additional papers are also approved using the criteria of the screening process and are marked with an asterisk in the table. The reasons for the decision to add these papers are reflected further in the discussion.

### 3.4. Data Analysis

To answer the research questions, information about the following main categories is extracted from each paper, as far as possible.

- Input Data Source:
  The input data source describes the origin of the sensor data or virtual force vector input information used for posture estimation.

- Estimation Method:
  The estimation method describes the method used to estimate human posture to give an overview of the most relevant methods.

- Application Scenario
  The application describes the scientific domain in which the study was conducted as well as the specific activities the participants performed in the studies.

Two large tables—one for ML-based posture estimation methods and one for DHM-based posture estimation methods—list all the information that is extracted for each paper in detail. To sum up all the information, the findings of the review are described for the main categories.

The required expertise and previous knowledge about the application context needed for using the methods cannot be quantified statistically from the review. Therefore, the results contain sections summarizing the estimation methods, input data sources and applications that form the basis of this review. The discussion section of this paper explores how the required expertise and knowledge about the application context affect the use of these methods in various domains, as elaborated by the authors.

A statistical evaluation of the results is presented using a Sankey diagram, which illustrates the relationship between the methods, models and corresponding data types used for human posture estimation. The Sankey diagram is created using the online tool SankeyMATIC (www.sankeymatic.com, Steve Bogart, accessed on 24 October 2023). Additionally, the diagram highlights the most commonly applied methods and models in different domains. To assess the novelty and current relevance of the methods found, the number of publications per year is plotted, with different colors representing the ML and DHM approaches in the period from 2000 to 2022. Therefore, the Software IBM SPSS Statistics Version 28.0. (IBM Corp. in Armonk, NY, USA) is used.

## 4. Results

The main results of our literature review are summarized, focusing on the main categories we aimed to analyze. Thereafter, a statistical evaluation of the results is shown. 

### 4.1. Input Data Sources and Sensors

The following summary is clustered into three sections. The first section covers the measurement principles that are used in the representative studies to record the data for posture estimation. Afterwards, the next section is about the sensor layouts of the hardware sensors before the difference between commercial and self-designed sensors is reflected.

#### 4.1.1. Measurement Principles

**Figure 4:**
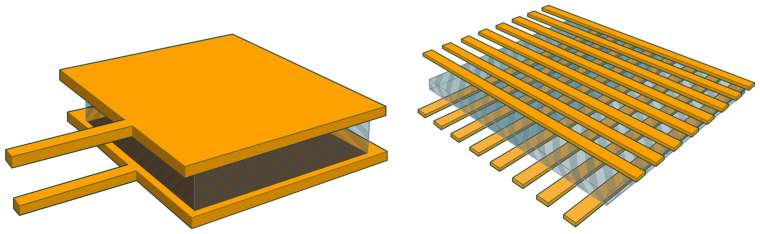
Sandwich structures of film pressure sensors consisting of a top and bottom copper layer connected by a piezoresistive ink: (**left side**) single-point sensor and (**right side**) sensor matrix [33] (Reprinted with permission from [33], 2023 Springer Nature).

The first screening criterion requires force or pressure data from hardware sensors or virtual force vectors as the main input parameters for human posture estimation. The sandwich structure of film pressure sensors consists of a top and bottom copper layer connected by a piezoresistive material (see Figure 4) [33].

The most commonly applied hardware sensors are thin film sensors that measure piezoresistive [13,16,34,35] or capacitive principles [15]. The sandwich structure of piezoresistive sensors consists of a top and bottom copper layer connected by a piezoresistive material (see Figure 4) [33]. Physical stress reduces the electrical resistance of the piezoresistive material and can be detected by an electronical measurement unit connected to the copper layers. The measurement principle allows a sensor thickness of up to 200 µm (FlexiForce A301, Tekscan Inc., Boston, MA, USA) and high flexibility. Capacitive sensors are filled with a non-conductive dielectric between the copper layers. Physical stress reduces the electrical capacity between both layers. Both measurement principles offer a similar measurement accuracy [36].

Additionally, load cells are also used in some studies to measure input data for posture estimation. Load cells differ in functionality and geometry from film pressure sensors. They typically use strain gauges or piezoelectric sensors and are not as thin as film sensors. Some load cells are capable of measuring more than just the force oriented perpendicular to the sensor. They also are capable of measuring forces in multiple directions and sometimes torques acting on the sensor. [37] Load cells are often more accurate than film pressure sensors. However, they tend to be more expensive and challenging in terms of the technical requirements for system integration [38].

Virtual force vectors are based on experience and knowledge about the application scenario. It requires a high expertise to define valid virtual sensors. They are found mostly in simulation studies. 

#### 4.1.2. Sensor Layout

The main difference that could be analyzed in the sensor layout is the difference between single-point film sensors, which can only measure the force or pressure value at one certain spot. The layout for load cells is comparable to the layout of single-point film sensors; also, they can partially measure the force in more than one dimension. The benefit of sensor matrixes is their ability to measure the force at multiple positions over a surface. On the other hand, the acquisition technology is more complicated for matrix sensors than for single-point sensors. Figure 4 shows the differences in the structure of a piezo-resistive single-point sensor and a piezo-resistive matrix sensor.

The selected sensor also influences the layout and positioning of the sensors in the application. The matrix sensors can mostly cover the entire relevant surface for pressure mapping. Single-point sensors have to be placed on the right spots to obtain all the relevant pressure information. Therefore, high expertise in the application is needed. Examples of different sensor layouts are given in Table 2.

#### 4.1.3. Commercial Hardware Sensors and Self-Designed Hardware Sensors

The commercial hardware sensors used in the reviewed studies are mostly developed for laboratory studies and are distributed by official partners. This allows other researchers to obtain the same equipment to replicate the study setup, reproduce the study and compare their results with it. Thus, the measurement quality of these sensors is standardized, which improves the comparability of the results. However, the sensors and additional measurement equipment can be quite expensive.

**Figure 5:**
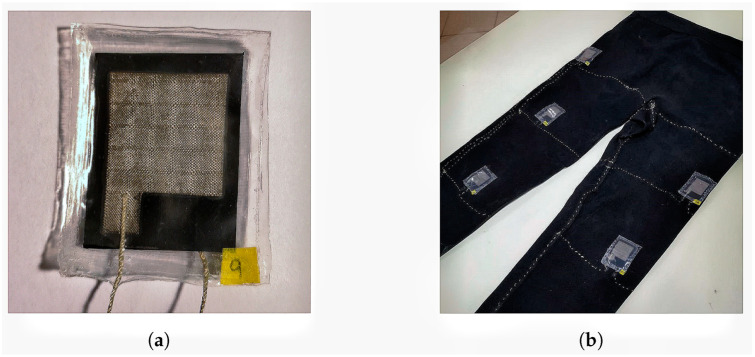
(**a**) Sample of the textile pressure sensors self-manufactured by [43].; (**b**) textile pressure sensors attached to expandable fabric pants [43] (Reprinted with permission from [43], 2023 MDP).

Self-designed sensors allow more individual adaptions through integration into products instead of commercial hardware sensors [42,43,44,45,46]. All the reviewed, self-designed sensors are film pressure sensors. They are mostly made of a pressure-sensitive film known by the brand names VELOSTAT (3M Company, St. Paul, MN, USA) and LINQSTAT (CAPLINQ Corporation, Ottawa, ON, Canada). Alternatives are offered by Matthies et al. [15], who developed their own capacitive sensors for a smart mattress, and by Milovic et al. [43], with their research on textile sensors, as shown in Figure 5.

### 4.2. Methods and Models for Human Posture Estimation

Human posture estimation methods can be divided into ML-based methods and DHM-based methods. ML-based methods work with only statistics, while DHM-based methods involve a lot of expert knowledge about the kinematics and dynamics of the human body. 

#### 4.2.1. Machine Learning Methods

In the reviewed papers, the performance and resource efficiency of different ML algorithms are compared under different constrains.

A general statement on the suitability of the algorithms for classification is not possible due to the different assumptions and constraints in the studies, as well as the lack of comparative values across studies. An overview of the most of used classification algorithms is given in Table 3. The resulting quality of the output data ranges from the simpler identification of body segments on a sensor mattress [13,47,48], to the assignment of predefined movement activities, to the classification of individual gait cycle phases directly related to leg joint angles during walking [43]. By using classification, only certain previously selected human postures can be recognized. These methods are well suited, e.g., to identify a critical situation during patient monitoring or analyze everyday activities. The continuous motion of a joint angle is not estimated using classification algorithms. Regression algorithms can also be used to estimate continuous joint angle motions. The regression model links the distribution and intensity of interaction forces in different situations to joint angles. Choffin et al. [12] use a K-nearest neighbor algorithm (kNN) and Clever et al. [49] use a convolutional neural network for posture estimation to obtain the continuous progress of a joint angle motion. Unlike other studies within this review, Rihar et al. [48] use an image recognition algorithm to evaluate the pressure map of a film sensor matrix to identify the different segments of a human body in a sleeping position. Image recognition is commonly used in AI applications. However, it is not popular for human posture estimation based on interaction forces.

The classification accuracies reach values above 90% for the most used algorithms (see Table 3). Data pre-processing is used in most of the studies to improve the quality of the data input to the algorithms. Filtering, e.g., using Gaussian filters or the sliding window effect, is used to reduce the noise in raw data. Different recording frequencies of sensors are also equalized, and sensors values can be fused, e.g., when defining measurement areas on a matrix in the pre-processing step.

Feature extraction and data pre-processing influence the time and resource efficiency and the accuracy of ML-based human posture estimation. The selection of feature extraction methods is contingent upon the sensor type and application scenario considerations of the different studies, e.g.,

- Center of pressure determination;
- Histogram of Oriented Gradients (HOG) analysis;
- Sliding window techniques;

Some studies embrace convolutional neural network (CNN), which obviate intricate, hand-crafted feature extraction. The most representative feature extraction methods are also listed in Table 3.

#### 4.2.2. Digital Human Models

**Figure 6:**
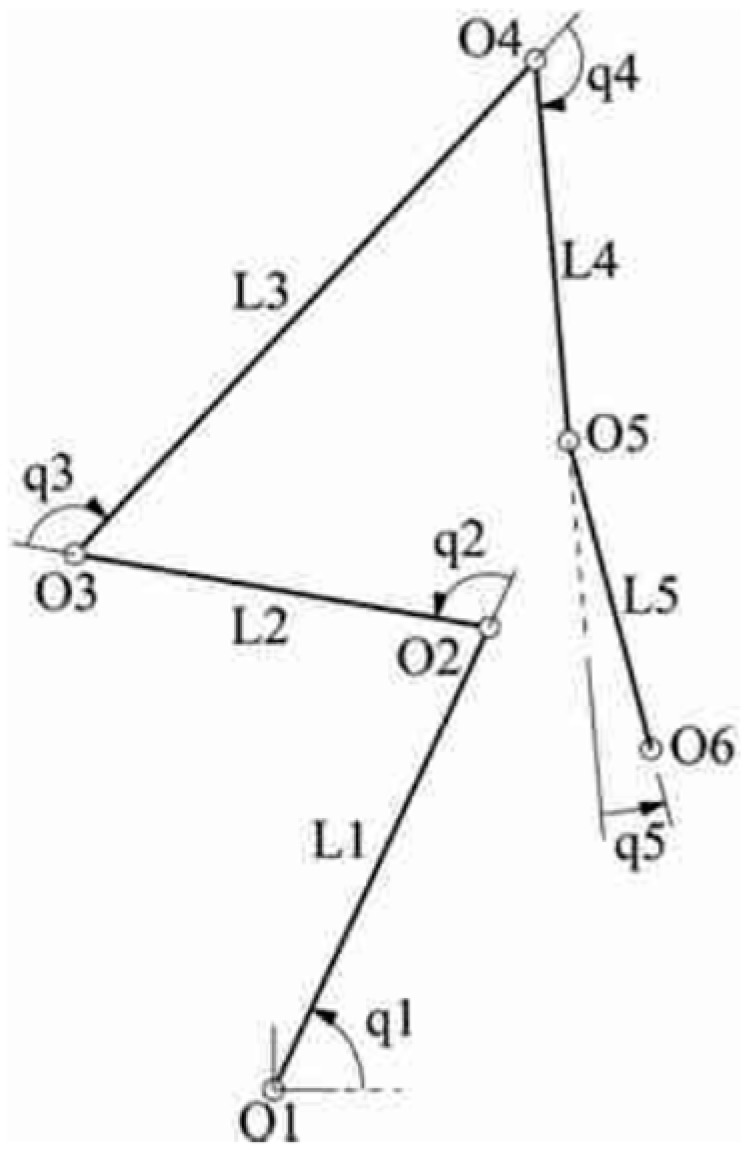
Visualization of the dynamic digital human model used by Rahmati and Mallakzadeh [78] for the posture estimation of a weightlifting sportsperson. (Reprinted with permission from [78], 2023 Elsevier B.V.)

DHM-based human posture estimation uses, unlike ML-based methods, knowledge of the kinematics and dynamics of the human body to extrapolate a human posture from force or pressure data. The DHMs used in the reviewed studies can be divided into two main categories. The first category consists of basic kinematic and dynamic models that focus on separating the segments of the human body. On the other hand, there are more complex and widely used DHMs that are commercially available or open-source and are often recognizable by their brand names. Based on the thermology of Demirel et al. [75], the latter ones can be further categorized into DHMs for 3D CAD and modeling and DHMs built as a finite element or multibody dynamics simulation. In most cases, posture estimation is realized by optimizing the joint angles to minimize a certain criterion, e.g., muscle activity or joint torques. An overview of the optimization criteria is given in Table 4:

Simple dynamic models are usually represented by rigid elements and joints with different degrees of freedom without spring or damper properties. An evaluation of these dynamic models is conducted via an inverse kinematic analysis. A typical dynamic model that is used for the posture estimation of a weightlifting sportsperson by Rahmati and Mallakzadeh [78] is visualized in Figure 6. 

**Figure 7:**
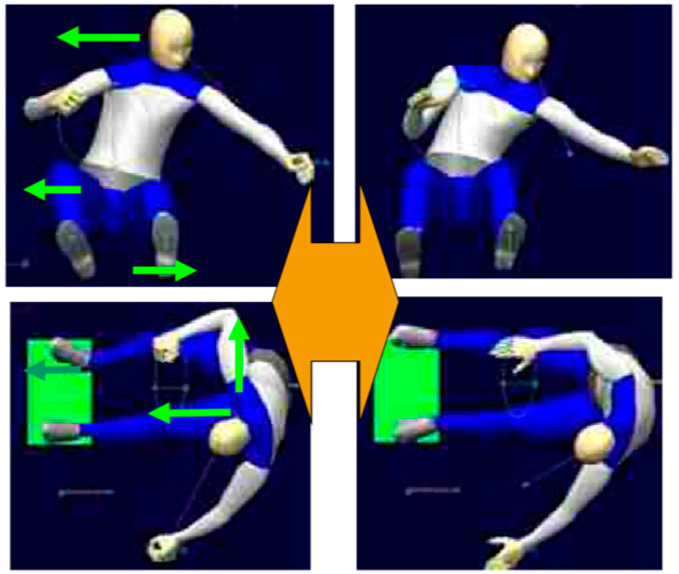
Using the RAMIS DHM for estimating the drivers posture closing a car door (**left**: force-based estimated posture, **right**: video-recorded real posture, green arrows displays the external forces for the FPE) [90].

Some of the studies use DHMs that were originally developed for early validation during the design process of ergonomic workshops and automotive cockpits. Thus, the concept of these models is a combination of a rigid dynamic model of the human body and a body shape model to provide a realistic impression of how humans can move inside the application environment [83]. In the reviewed studies, the SANTOS model (SantosHuman Inc., Coralville, IA, USA) is mainly used to perform inverse kinematic analyses [77,84,85]. Its evaluation is similar to that of simple kinematic models but is supported by the user interface and a realistic representation of the human body. The JACK model (Siemens Industry Software Inc., Plano, TX, USA) [86], the RAMSIS model (Human Solutions GmbH, Kaiserslautern, Germany) [87,88] and the 3DSSPP model (VelocityEHS Inc., Chicago, IL, USA) [89] are used as dynamic models similar to the SANTOS model [88], but also as body shape models, e.g., to calculate the contact surface between the human and a pressure-sensing object. A use case for the RAMIS DHM is shown in Figure 7.

The DHMs in the “finite elements and multibody dynamics DHM” category represent the structure and the interaction of the human body in the most detailed, but also most complex, way. The studies for human posture estimation are dominate by the use of musculoskeletal models. Musculoskeletal models are multibody dynamic simulations built up from the bones of the human skeleton as rigid bodies and the muscles as dynamic connections. In many studies, the commercial software AnyBody (AnyBody Technology A/S, Aalorg, Denmark) is used to calculate muscle activity and optimize the body posture for minimal muscle activity [79].

### 4.3. Application Scenarios

Human posture estimation based on interaction forces serves as an automated alternative when traditional camera-based posture estimation is infeasible or more costly to install. This is particularly useful in scenarios where camera installation may limit mobility or when the use case involves a virtual posture estimation. In such cases, the main input condition is the impact of an external force on the human body. The main application domains of the studies investigated are healthcare—especially for patient monitoring—[13,22,35,39,46,47,48,61,64,71], sleep research [45,49,91,92,93], sports science [37,76,94], product [84] and automotive design [34,87,88] and the sub-domains of ergonomics—industrial ergonomics [12,50,77,80,82,86,89,95,96], gait analyses [43,51,65,85,97] and sitting ergonomics [14,16,21,44,54,69,72,98,99,100,101]. The context of the application scenarios also influences the estimation methods that are best to use for the posture estimation. For example, the application scenario can limit the training data that can be generated for the ML-based methods. Further, the application scenarios also influence what kind of sensor can be used and where it should be placed to measure the force data.

### 4.4. Findings

**Figure 8:**
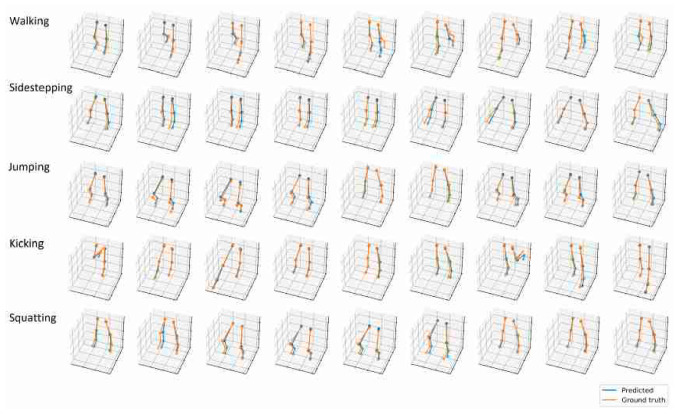
Comparison of estimated lower-body motion and a motion-captured ground truth [102] (Reprinted with permission from [102], 2023 IEEE Xplore).

The findings and results from the reviewed papers can be categorized into several areas, including the design and integration of sensor hardware into products for specific applications, testing the performance of new ML algorithms for posture estimation and comparing them, applying and testing validated ML algorithms for new applications and input data and improving existing DHMs for human posture estimation. Figure 8 shows the result of lower-body posture estimation nearly fitting perfectly to the ground truth data.

### 4.5. Sankey Diagram

**Figure 9:**
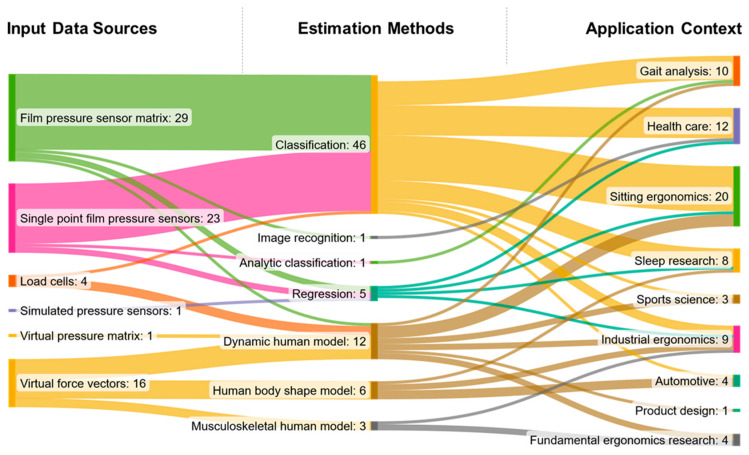
Sankey diagram showing the relations between input data sources, estimation methods and applications, including the number of studies found.

The Sankey diagram (Figure 9) shows the connections between the input data sources, the estimation methods and the application scenarios for which the estimation methods are used. It also shows the percentage of times each data source, method, and application are used in the reviewed studies. When dividing studies into those with ML-based methods and DHM-based methods, the classification algorithms favor ML-based estimation methods, while the basic dynamic human models favor the DHM-based estimation methods. Furthermore, film pressure sensor matrices and single-point film pressure sensors are the main input data sources for the classification, and virtual force vectors are mainly used as input for DHM-based posture estimation. The utilization of the different methods is evenly distributed among the different applications. Only in the healthcare domain—especially for patient monitoring—classification algorithms dominate. The main applications are patient monitoring in the healthcare domain and product optimization in various subdomains of ergonomics. 

### 4.6. The Novelty and Relevance of Human Posture Estimation

**Figure 10:**
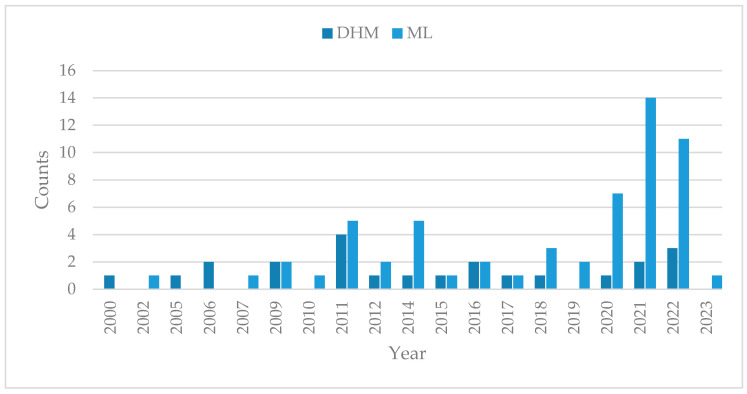
Histogram of published studies on force-based human posture estimation, divided into machine learning (ML)-based estimation methods and digital human model (DHM)-based estimation methods, over the years.

The histogram in Figure 10 shows the emergence of DHM-based methods for human posture estimation, with the investigation of these methods starting in the early 2000s. There has been a notable increase in the number of publications on ML-based methods since 2007, indicating a growing interest in recent years. In contrast, the number of publications on DHM-based methods has remained relatively constant over the years.

#### Datailed Information about the Analysed Studies

A detailed analysis of the representative studies serves as the basis for the summarized findings below and is presented in the following tables. Within Table 5, the data includes input data sources, estimation algorithms, performance evaluations, data preprocessing and feature extraction, and application scenarios derived from studies using machine learning-based methods. Meanwhile, Table 6 presents information from studies using digital human models (DHMs), including details on input data sources, the specific DHM, optimization criteria, and application scenarios.

## 5. Discussion

As motivated in the beginning, the research on human posture estimation based on interaction forces is characterized by a high level of interdisciplinarity extending from computer science and sensor development to product design, ergonomics and health care. This interdisciplinarity challenges researchers to obtain an easy overview from the literature of the existing methods and models and the boundary conditions for their application. To close this knowledge gap and support the utilization of the methods of human posture estimation based on interaction forces, the research questions of this systematic literature review are divided into two:

1. Extracting the used input data sources and estimation methods from the literature.
2. Evaluating the types of application scenarios and previous expert knowledge used in the reviewed studies.

The first aspect can be answered through a data analysis of the review’s results, as illustrated by the Sankey diagram in Figure 8. Summarizing the results, the main methods for human posture estimation are classification algorithms when ML-based methods are used and inverse kinematic analyses of simple dynamic models when DHM-based methods are used.

Classification algorithms mainly use input data from thin-film pressure sensors, which offer advantages such as flexibility and seamless integration into existing products. On the other hand, DHM methods use virtual force vectors, which require a comprehensive understanding of the use case. Nevertheless, the final results should be validated by an experimental study. Such validation is conducted in the studies by Davoudabadi Farahani et al. [79] and Mao et al. [99].

The second aspect of the research questions cannot be answered only by an analysis of the review data. A specific interpretation of the application scenarios is necessary. Additionally, the histogram is helpful for understanding the necessary previous expert knowledge. The main findings of our systematic literature review are illustrated in Table 7 and discussed in the following to support researchers in selecting the best methods and input data sources for their application.

With the advent of AI research and standard ML algorithms for common applications of data processing, ML algorithms are also used for force-based human posture estimation. The principles of the ML-based methods can be understood as a virtual sensor [112]. They use data from hardware sensors, mostly force or pressure sensors, and apply previously trained ML models to estimate human posture. These algorithms provide established frameworks, efficient computations and well-documented methodologies, making them suitable for a wide range of applications. Many studies in the literature compare the efficiency and resulting quality of different ML algorithms for classification. Due to the divergent conditions in the studies, in an evaluation, the best algorithm for human posture estimation based on interaction forces is not obvious. The most often used algorithms are CNN, kNN, SVM, RF, and NB algorithms. Data processing using ML algorithms does not require specific knowledge about the physiology of the human body. Although more advanced techniques and algorithms are being developed, the use of standard algorithms remains a practical choice due to their ease of implementation, computational efficiency and performance. When using ML algorithms, the training data are obtained from hardware sensors, which record data during experimental studies similar to the situations in which human posture should be estimated. This required more effort for the adaptation of these methods to a new application than in the simulation environment of a DHM. Due to the need for sensor data as the input for the ML algorithms, research on hardware sensors and their integration into everyday products becomes increasingly important. It also demonstrates a chance to apply ML-based methods for automated utilization in sensor-integrated products. In the current state of research, the majority of published studies focus on classification algorithms with relatively simple class definitions. These are mostly various movement activities or specific phases of a gait cycle. However, there are few works that use ML algorithms to continuously estimate one or more human joint angles. Further research is needed on this topic to provide an established procedure for the utilization of regression algorithms for human posture estimation based on interaction forces.

The use of DHMs began in the early 2000s and forms the baseline for FPE [88]. Most often, muscle activation and joint forces are computed to analyze the physical stress of a human subject. Reversing this idea, DHM-based posture estimation optimizes physical stress by adjusting human posture. The valid application of these DHM-based methods requires specific expert know-how about the physiology of the human body in the form of a DHM and comprehensive knowledge about boundary conditions and external influences on humans for the specific use cases. As a result, only experts who are very familiar with DHMs can use them properly. These experts come from the fields of ergonomics and medical research. Simulation-based posture estimation offers several advantages, including its applicability to non-physical existing scenarios and the ease of adapting human model properties or external influences. Also, these methods eliminate the need for conducting physical experiments, resulting in faster and more cost-effective processes. However, an online posture estimation is not known to exist in the literature so far. Due to the highly required expertise for the utilization of DHMs and the expert knowledge needed for their application, an online estimation process and product integration are not expected soon.

As mentioned in the methods for the review process, a challenge we encountered was the lack of consistent terminology used in the research community for human posture estimation based on interaction forces. To address this issue, we clustered different synonymous keywords for the search string. Nevertheless, not all papers involving human posture estimation based on interaction forces can be covered by this literature research. In particular, studies using DHMs are difficult to find without searching for the specific DHM method names, although some were added manually based on the authors’ knowledge. We prioritized the dominating keywords “posture” and “estimation” for the terminology of the review paper. To gain a more comprehensive understanding, it is helpful to search for similar review papers, e.g., on motion capture in general [9] or the various specifications and applications of DHMs [75]. In this way, it is possible to find publications using alternative terminologies. However, methods for force-based human posture estimation are not the focus of these papers. Thus, it is left to the reader to compare the different methods and evaluate their suitability by themselves.

Due to this inconsistent use of terminology, there is no suitable search string that identifies all known papers on studies of FPE. Therefore, we decided to include the papers we were aware of from previous searches in the results. While this approach differs from a classical structured literature review, we chose it in order to get closer to a complete overview of the published methods.

## 6. Conclusions

This systematic literature review, according to PRISMA 2020 [30], provides an overview of the relevant estimation methods, dominated by classification algorithms and the posture optimization of digital dynamic human models. As a guideline for the existing methods, the required expertise and information on the application context are extracted from the reviewed studies. The data used for the posture estimation come from experimental studies using thin flexible film pressure sensors, and in virtual simulations, from force vectors. The required expertise for the utilization of a method and the know-how about the application context tend to increase the resultant benefits, as illustrated in Table 7. DMH-based methods can be performed in virtual simulations without relying on experimental studies, but they require a more specific understanding to build and apply the models specific to the use case. On the other hand, conventional ML methods can be used to estimate activity classes or human posture when sufficient experimental training data are available, with less need for expert knowledge. As part of the ongoing research in this area, it is worth noting that the review has highlighted the remarkable performance achieved by classification algorithms. However, the inherent limitation of classifying a predefined set of postures has become apparent. To bridge the gap and achieve results on par with established methods such as camera-based posture estimation and IMU sensors, the use of regression algorithms becomes imperative. Interestingly, a small number of studies have ventured into this area, investigating joint angles in gait analysis and upper body alignment during sitting. These initial forays offer a promising glimpse into the potential of regression algorithms in posture assessment. However, it is clear that more extensive research is needed to thoroughly explore and validate the utility of such approaches. 

Furthermore, an evaluation of the application scenarios and previous expert knowledge was conducted based on the results of the review to further assist researchers in the selection of the best estimation methods and input data sources for their application. The evaluation of the application context showed that ML-based methods consistently perform well in tasks such as sleep and sitting position recognition, as well as gait analysis. However, DHMs are preferred for manual tasks. There is a noticeable gap in research regarding the potential suitability of ML-based applications for posture assessment in manual tasks. Further research in this area is warranted to explore its feasibility. The review also has some limitations, including the non-standardized terminology of the keywords and the inclusion of studies focusing on activity recognition, which may not necessarily count towards posture estimation.

## Figures and Tables

**Table 1:** Clustered keywords selected as search string for this literature review.

Human	Posture	Estimation	Force	Method
	pose	prediction	pressure	model
		classification	load	
		recognition	weight	

**Table 2:** Examples of sensor layouts for measuring the contact forces for force-based human posture estimation in different application scenarios.

Application Scenario	Positioning of Single-Point Sensors	Layout of Sensor Matrix
**Sensor mattress (health care + sleep research)**	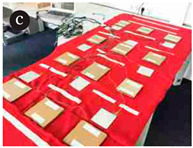 DIY sensor mattress with capacitive single-point sensors [15]	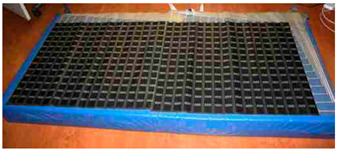 Commercial piezorestive sensor matrix on a sleeping matrix [39] (Reprinted with permission from [39], 2023 IEEE Xplore)
**Sensor insoles** **(gait analysis + sport science)**	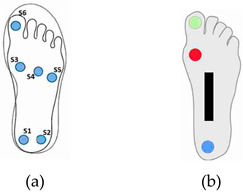 Placement of single-point FSR sensors (circles) at an insole with (a) 6 FSR sensors [12] and (b) 3 FSR sensors and 1 bend sensor (black rectangle) [40] (Reprinted with permission from [12], 2023 MDPI, and [40], 2023 IEE Xplore)	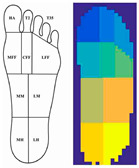 Sensor layout of an F-Scan insole sensor matrix with 954 measure points [41] (Reprinted with permission from [41], 2023 Spriger)
**Sensor chair (sitting ergonomics + automotive design)**	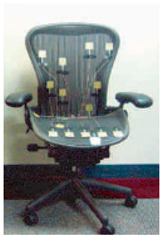 Positions of DIY single-point sensors on a smart chair [14]	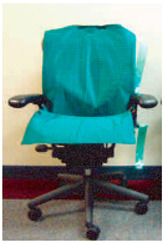 Application of two Tekscan Conformat sensors on an office chair [14]

**Table 3:** Overview of the most often studied classification algorithms for force-based human posture estimation.

Classification Algorithm	Classification Accuracy	Feature Extraction Methods	Relevant Studies
**Support vector machine (SVM)**	70% [34]–99.79% [50]	Raw data [51], statistics [50,52], sliding window [40,50], HOG [39,46]	[14,16,22,34,39,40,41,46,50,51,52,53,54,55,56,57,58,59,60]
**K-nearest neighbor (kNN)**	78% [34]–98.52% [22]	Raw data [61,62], statistics [50], sliding window [50]	[13,22,34,40,41,47,50,54,55,61,62,63,64,65] *used for regression:* [12]
**Convolutional Neural Networks (CNNs)**	84.80% [21]–99.84% [66]	Mostly automated feature extraction by CNN network	[21,29,35,63,66,67,68,69,70,71,72] *used for regression:* [29,49]
**Random forest (RF)**	52.2% [34]–100% [40]	Sliding window [40], statistics [60]	[34,40,53,54,57,60,63,73,74]
**Naïve Bayes (NB)**	40.09% [22]–84.33% [16]	Mostly filtered raw data	[16,22,34,44,54,63,73]
**Decision Tree (DT)**	76.79% [16]–98.10% [50]	Statistics [50] Sliding window [41,50]	[16,41,50,53,54,63,73]

**Table 4:** Overview of the optimization criteria for DHM-based human posture estimation.

Optimization Criterium	Digital Human Models	Relevant Studies
Reduction in joint torque	3D dynamic human model	[76,77,78]
Reduction in muscle effort	Musculoskeletal human model	[79,80]
Hypothesis of quasi-static equilibrium	Dynamic human model	[81]
Reduction in joint fatigue	2D three-compartment controller fatigue model	[82]

**Table 5:** Summary of relevant studies for ML-based human posture estimation and the analysis of the used input data sources, estimation algorithms, acquired accuracy, data pre-processing/feature extraction and application scenarios (* marks the extra-added reports after the structured literature review); the best performing estimation algorithm for each study is highlighted **bold**.

Author	Year	Input Data Source	Estimation Algorithm	Estimation Performance	Data Pre-Processing/Feature Extraction	Application Scenarios
**Adami et al. [92]**	2011	Single-point film pressure sensors: AG100 C3SH5eF (Scaime™, Juvigny, France)	Classification: **Gaussian mixture models**	Classification rates for individual subjects: from 76.7% to 95.3% Over all classification rate: up to 84.6%	Data pre-processing: Analysis of the trajectory of the body’s center of mass Feature extraction: (1) the Euclidean distance between initial and end points of the trajectory (2) the trajectory length and (3) the variance of the trajectory in the y-direction perpendicular to the sleeper’s body axis	Sleep research: Classification of the movement type lying in a bed
**Adami et al. [91]**	2014	Single-point film pressure sensors: 6 AG100C3SH5eU (Scaime™, Juvigny, France)	Classification: **Linear discriminant function**	Classification rate: 96.9% Spearman’s correlation coefficient between the periodic leg movement indexes estimated by the system and those obtained from a polysomnogram is 0.927	Data pre-processing: trajectory of the center of pressure (CoP) Feature extraction: (1) the Euclidean distance between initial and end points of the trajectory (2) the trajectory length, and (3) the variance of the trajectory in the y-direction perpendicular to the sleeper’s body axis.	Sleep research: Detection of periodic limb movement events
**Ahmad et al. [53]**	2021	Single-point film pressure sensors: Large-area screen-printed sensor (16 pressure-sensing elements, covering an effective sitting area of 505 cm^2^	Classification: k-nearest neighbors (kNN), support vector machine (SVM), random forest (RF), decision tree (DT) and **light gradient boosting machine (LightGBM)**	Classification accuracy: kNN: 98.27% RF: 98.07% SVM: 95.00% DT: 97.81% **LightGBM: 99.03%**	-	Sitting ergonomics: Identification of sitting posture abnormalities on a wheelchair
**Aminosharieh Najafi et al. [21]**	2022	Single-point film pressure sensors: 8 force-sensing resistors (FSRs, unspecified)	Classification: Feedforward artificial neural network, multilayer perceptron (MLP), convolutional neural network (CNN), bidirectional long short-term memory (Bi-LSTM), CNN-LSTM (CNLSTM), convolutional LSTM (CVLSTM), and **echo memory network (EMN)**	Classification accuracy: MLP: 90.83% CNN: 86.99% LSTM: 88.71% BDLSTM: 88.98% CNLSTM: 89.14% CVLSTM: 89.97% **EMN: 91.68%**	Data pre-processing: Smoothing motion raw data using Savitzky–Golay filter	Sitting ergonomics: Classification of sitting postures
**Antwi-Afari et al. [50]**	2018	Film pressure sensor matrix: Wearable insoles ”OpenGo” (Moticon GmbH, Munich, Germany), 3D MEMS-accelerometer (BMA 150, Bosch Sensortech GmbH, Reutlingen, Germany)	Classification: Artificial neural network (ANN), decision tree (DT), K-nearest neighbor (kNN), and **support vector machine (SVM)**	Classification accuracy (10-fold cross-validation): ANN: 97.60% DT: 98.10% KNN 98.60% **SVM: 99.79%**	Data pre-processing: Sliding window segmentation (0.32 s) with 50% data overlap Time-domain features: (1) Mean pressure; (2) Variance; (3) Maximum pressure; (4) Minimum pressure; (5) Range; (6) Standard deviation Frequency-domain features: (1) Spectral energy (2) Entropy Spatial temporal features: (1) Pressure time integral	Industrial ergonomics: Classification of awkward working positions
**Antwi-Afari et al. [95]**	2022	Film pressure sensor matrix: Wearable insoles ”OpenGo” (Moticon GmbH, Munich, Germany), 3-axis gyroscope (MEMS LSM6DSL, ST Microelectronics, Plan-les-Ouates, Swiss)	Classification: RNN-based deep learning models: long short-term memory (LSTM), Bi-LSTM, and **gated recurrent units (GRU)**	Classification accuracy: LSTM: 97.99% Bi-LSTM: 98.33% **GRU: 99.01%** Training time: LSTM: 31 min Bi-LSTM: 56 min **GRU: 54 min**	Automated feature extraction by RNN	Industrial ergonomics: Classification of awkward working positions
**Bhatt et al. [67]**	2021	Simulated pressure sensors: A synthetic portion of the bodies at rest data set	Classification: **Convolutional neural network (CNN)** with a ResNet backbone	Recall score: up to 99%	Automated feature extraction by CNN algorithm	Sleep research: Sleeping posture detection
**Beltrán-Herrera et al. [46]**	2014	Film pressure sensor matrix: Low-cost flexible array pressure sensor with 448 units	Classification: Support vector machine (SVM)	-	Feature extraction: Histogram of Oriented Gradients (HOG) descriptor	Health care: Body classification in lying state
**Bourahmoune et al. [73]**	2022	Single-point film pressure sensors: LifeChair (Pressure-sensitive desk chair with 9 DIY piezoresistive film pressure sensors)	Classification: Decision tree classification and regression trees (DT-CART), **random forest (RF)**, k-nearest neighbors (kNN), linear regression (LR), linear discriminant analysis (LDA), naïve Bayes (NB) and neural network multilayer perceptron (MLP).	Classification accuracy: **RF: 97.09%** DT-CART: 96.19% kNN: 92.13% NN (MLP): 80.09% LR: 5367% LDA 53.16% NB: 41.71%	Feature extraction: Pressure distribution Additional feature: Body mass index (BMI)	Sitting ergonomics: Posture detection for desk working scenarios
**Casas et al. [61]**	2018	Film pressure sensor matrix: Pressure sensors arranged horizontally and equidistantly, covering an area of 2 m × 1 m.	Regression: Fusion of k-nearest neighbor (kNN)	Mean absolute errors (MAE): Joint angle: 17.5–25.4° Joint position: 9.76–14.02 cm	Feature extraction: Each sensor represents a stand-alone feature	Health care: Patient monitoring
**Choffin et al. [12]**	2021	Single-point film pressure sensors: 6 FSR sensors (FlexiForce A301, Tekscan Inc., Boston, MA, USA)	Regression: K-nearest neighbor (kNN)	Motion classification accuracy: 93.6% Angle Regression accuracy: 87.4%	-	Industrial ergonomics: Lowering risk of musculoskeletal injuries
**Clever et al. [49]**	2020	Simulated pressure sensors: Co-simulation: FleX and DART	Regression: Convolutional neural network (CNN)	Average pose estimation error: <5 cm	Automated feature extraction using CNN algorithm	Sleep research: Estimation of human sleeping posture
**Cruz-Santos et al. [39]**	2014	Film pressure sensor matrix: 2 pressure sensor arrays with 16 × 16 units in an area of 1108 × 554 mm (SensingTex, Barcelona, Spain)	Classification: Support vector machine (SVM)	Classification accuracy: **Raw Data: 99.70%** HOG: 98.70% SIFT: 98.95%	Feature extraction: **(1) Raw data; ** (2) Histogram of Oriented Gradients (HOG) descriptor; (3) Scale Invariant Feature Transform (SIFT) descriptor	Health care: Patient monitoring
**Cun et al. [54]**	2021	Film pressure sensor matrix: Body pressure measurement system (BPMS) (Tekscan Inc., Boston, MA, USA) (2 Sensors)	Classification: K-nearest neighbor (kNN), **support vector machine (SVM),** random forest (RF), decision tree (DT) and naïve bayes (NB)	Classification accuracy: kNN: 82.65% **SVM_RBF_: 89.26%** SVM_linear_: 83.47% RF: 85.95% DT: 77.69% NB: 79.34%	Algorithm specific features; no detailed description	Sitting ergonomics: Classification of sitting postures/activities
**Diao et al. [45]**	2021	Film pressure sensor matrix: Low-cost flexible array pressure sensor with 32 × 32 units based on Velostat^®^ film (3M Company, St. Paul, MN, USA)	Classification: Deep residual network (ResNets)	Classification accuracy: short-term: 95.08% overnight sleep study: 86.35%	No hand-craft feature extraction	Sleep research: Estimation of human sleeping posture
**Diao et al. [55]**	2021	Film pressure sensor matrix: Low-cost flexible array pressure sensor with 32 × 32 units based on Velostat^®^ film (3M Company, St. Paul, MN, USA)	Classification: **K-nearest neighbor (kNN)**, support vector machine (SVM)	Classification accuracy: **kNN: 79.02%** SVM: 78.19%	Feature extraction: (1) Pressure-covered area of the mat; (2) Local pressure coverage area ratio; (3) Local pressure value ratio; (4) Left–right symmetry; (5) Left–right balance	Sleep research: Estimation of human sleeping posture
**Diao et al. [103]**	2022	Film pressure sensor matrix: Low-cost flexible array pressure sensor with 32 × 32 units based on Velostat^®^ film (3M Company, St. Paul, MN, USA)	Classification: Self-developed low-cost estimation algorithm, LeNet-1, 2 Conv + 3 FC, **ResNet18**	Classification accuracy: New estimation algorithm: 98.3% LeNet-1: 99.1% 2 Conv + 3 FC: 99.2% **ResNet18: 99.4%**	Data pre-processing: (1) Threshold filtering; (2) Gaussian filtering; (3) Adjacent affected noise removal Feature extraction: (1) The Euclidean distance between initial and end points of the trajectory	Sleep research: Estimation of human sleeping posture
Djamaa et al. * [40]	2020	Single-point film pressure sensors: 3 x FSR 402 (Interlink Electronics, Inc., Shenzhen, China); Additional Sensors: Bend sensor + IMU MPU 6050 (TDK InvenSense, Inc., San Jose, CA, USA)	Classification: Support vector machine (SVM), k-nearest neighbors kNN (k = 5), stacked **random forest (RF), MultiBoostAB with RF (MB-RF)** and MultiBoostAB with logistic model tree (MB-LMT)	Classification accuracy: SVM: 98.20% (8 s) **RF: 100% (7 s and 8 s)** **MB-RF: 100% (7 s)** MB-LMT: 96.59% (10 s) Stacked: 91.51% (3 s) kNN: 87.91% (3 s) Response time: MB-RF: 0.4589 ms RF: 1.6113 ms SVM: 0.7272 ms	Feature extraction: Segmentation using a sliding window procedure with a fixed-length window	Gait analysis
**Feng et al. [104]**	2016	Fiber optical pressure Sensor	Classification	Classification accuracy: 96% to 100%	-	Fall detection
**Fulk et al. [56]**	2011	Single-point film pressure sensors: 5 force-sensitive resistors (FSR, Interlink Inc, Camarillo, CA, USA) Additional Sensors: 3-dimensional accelerometer	Classification: Support vector machine (SVM)	Classification accuracy: 99.91% to 100%	Feature extraction: (1) nonoverlapping 2 s epochs (2) normalized on a scale of [0, 1]	Gait analysis
**Gao et al. [66]**	2022	Single-point film pressure sensors: Mattress with 8192 force-sensitive resistors (FSR, Interlink Inc, Camarillo, CA, USA)	Classification: AlexNet, 2-layer CNN, VGG and **MatNe**	Classification accuracy: AlexNet: 93.67% 2-layer CNN: 94.09% VGG: 98.59% **MatNe: 99.02%** Response time:AlexNet: 0.094 s 2-Layer CNN: 0.043 s VGG: 0.56 s MatNe: 0.095 s	Automated feature extraction	Sleep research
Gelaw et al. * [57]	2022	Film pressure sensor matrix: Intelligent Interfaces and Interaction research unit of Fondazione Bruno Kessler (FBK, Trento, Italy).	Classification: Random forest (RF), Gaussian naïve bayes, logistic regression, support vector machine (SVM) and **deep neural network (DNN)**	Classification accuracy: RF: 82% GNB: 88% SVM: 75% LR: 81% DNN: 93%	Feature extraction: (1) 64 sensor features (2) center of mass of the 32 sensors of the seat and back (3) features for each of the seat and back sensors, dividing the given 32 sensors into four quadrants and edges	Sitting ergonomics: Estimation of sitting posture during work on a PC
Goldstein et al. * [29]	2020	Film pressure sensor matrix: TekScan BPMS (Tekscan Inc., Boston, MA, USA)	Classification and Regression: Convolutional neural network (CNNs);	Classification accuracy: 95% Regression accuracy: average marker position error: 8.84 cm	Automated feature extraction by CNN algorithm	Sitting ergonomics: Slouch detection
**Grimm et al. [47]**	2011	Film pressure sensor matrix: XSensor X3 PX100:26.64.01 mattress (XSensor, Calgary, AB, Canada).	Classification: K-nearest neighbor (kNN)	Classification accuracy: 96.0%	-	Health care: Patient monitoring
**Grimm et al. [13]**	2012	Film pressure sensor matrix: Pressure-sensing mattresses	Classification: K-nearest neighbor (kNN)	Classification accuracy: 95.5%	-	Health care: Patient monitoring
**Harada et al. [105]**	2002	Single-point film pressure sensors: Mattress with 210 force-sensitive resistors (FSR, Interlink Inc, Camarillo, CA, USA)	Classification	-	-	Health care: Patient monitoring
**He et al. [68]**	2022	Film pressure sensor matrix: Thin-film array pressure sensor, which is made by transferring nanometer force-sensitive materials, silver paste and other materials to a flexible thin-film substrate, via a precision printing process	Classification: Convolutional neural network (CNN)	Classification accuracy: Improved ResNet: 99.86% ResNet: 90.99% VGG16: 81.26% MobileNet: 78.30%	Data pre-processing: Cross-mean filtering Extreme value processing Feature extraction: (1) Improved ResNet (2) ResNet50 (3) VGG16 (4) MobileNet	Sitting ergonomics: Detection of sitting postures
**Hsia et al. [58]**	2009	Single-point film pressure sensors: Two types of FSR from Interlink Electronics [10] were used: part no. 402 (0.5″ circle) and part no. 408 (24″ trimmable strip).	Classification: Support vector machine (SVM)	Classification accuracy: PCA + SVM: ≤75% Raw data + SVM: ≤92% **Descriptive statistics + SVM: ≤95%**	Feature extraction: Principal component analysis (PCA), **descriptive statistics (mean, variance, standard deviation, root-mean squared features, etc.)**	Sleep Research: Sleeping posture classification
**Hu et al. [35]**	2021	Film pressure sensor matrix: Low-cost flexible array pressure sensor with 32 × 32 units based on Velostat^®^ film (3M Company, St. Paul, MN, USA)	Classification: Convolutional neural network (CNN)	Classification accuracy: (1) Standard training-test method: 84.80%; (2) Subject-specific method: 91.24%	Automated feature extraction using CNN algorithm	Health care: Patient monitoring
**Jeong [62]**	2021	Single-point film pressure sensors: 6 force-sensing resistors (FSR) Additional sensors: 6 distance sensors	Classification: k-nearest neighbor (kNN)	Classification accuracy: (1) Pressure sensors only: 59%; (2) Distance sensors only: 82%; **(3) Mixed sensor: 92%**	Feature extraction: (1) pressure sensors only; (2) Distance sensors only; **(3) Mixed sensor**	Sitting ergonomics: Classification of sitting postures
**Lee et al. [69]**	2022	Film pressure sensor matrix: 64 (8 × 8) FSRs (TechStorm, Seoul, Republic of Korea)	Classification: Convolutional neural network (CNN)	Classification accuracy: 99.66%	Automated feature extraction using CNN algorithm	Sitting ergonomics: Classification of sitting postures/activities
**Li et al. [96]**	2020	Film pressure sensor matrix: Pressure-sensing floor	Classification: Convolutional neural network (CNN), support vector machine (SVM), k-nearest neighbor (kNN), random forest (RF), decision tree (DT), naïve bayes (NB), BP neural network classifiers and **CSK-DS-fusion algorithm** (CNN + SVM + kNN + D-S)	Classification accuracy with threshold filter: CNN: 96.28% SVM: 94.45% kNN: 91.38% RF: 92.16% DT: 89.23% NB: 83.37% BP: 83.28% **CSK-DS: 99.96%**	Data pre-processing: Threshold filtering Gaussian filtering	Industrial ergonomics: Standing posture estimation
**Li et al. [63]**	2020	Film pressure sensor matrix: Pressure-sensing floor	Classification: Convolutional neural network (CNN),	Classification accuracy 96.6%	-	Industrial ergonomics: Standing posture estimation
**Liu et al. [70]**	2019	Film pressure sensor matrix: Flexible piezoresistive array sensor with pixel size of 64 × 32 and single sensor with maximum test pressure of 500 N	Classification: Convolutional neural network (CNN)	Classification accuracy: CNN: 97.2%	Automated feature extraction by CNN algorithm	Sleep Research: Sleeping posture classification
**Ma et al. [22]**	2017	Single-point film pressure sensors: 12 force-sensitive-resistor pressure sensors (FSR-406, Interlink Inc., Camarillo, CA, USA)	Classification: **J48 decision tree (J48)**, support vector machine (SVM), multilayer perceptron (MLP), naïve bayes (NB; default + BayesNet), and k-nearest neighbor (kNN)	Classification accuracy: **J48: 99.48%** SVM: 79.08% MLP: 95.5% Naïve Bayes (default): 40.09% BayesNet: 94.53% kNN (k = 1): 98.53% kNN (k = 5): 98.52%	Algorithm-specific feature extraction	Health care: Patient monitoring in a wheelchair
**Matar et al. [106]**	2020	Single-point film pressure sensors: a matrix of 64 × 27 textile-made piezoresistive pressure sensors	Classification: Feed-forward artificial neural network (FFANN)	Classification accuracy: 97.9%	Feature extraction: (1) Histogram of Oriented Gradients (HOG) descriptor (2) Local Binary Patterns (LBP)	Sleep Research: Sleeping posture classification
Matthies et al. * [15]	2021	Single-point film pressure sensors: DIY-sensor mat: pressure tiles (binary switches) + capacitive sensor (continuous data stream).	Classification: Random forest (RF)	Classification accuracy: 85.02%	-	Health care: Patient monitoring: bed-exit intention detection, pressure ulcer prevention and sleep apnea mitigation
**Meyer et al. [44]**	2010	Film pressure sensor matrix: Textile pressure sensor (new sensor), Tekscan Conformat (Tekscan Inc., Boston, MA, USA)	Classification: Naïve bayes (NB)	Classification accuracy: Up to 82%	Data pre-processing: With and without hysteresis compensation Feature extraction: (1) Sensor value from each sensor element; (2) Center of force; (3) Pressure applied to 4 and 16 equally aggregated areas of the seating area	Sitting ergonomics: Classification of sitting postures/activities
**Merry at al. [41]**	2021	Film pressure sensor matrix: F-Scan pressure measurement insoles (Tekscan Inc., Boston, MA, USA)	Classification: **Support vector machine (SVM)**, decision tree (DT), discriminant analysis (DA), and k-nearest neighbors (kNN)	Classification accuracy: **SVM: 89.73%** DT: 89.45% DA: 83.86% kNN (k = 1): 84.87%	Data pre-processing: Subdivision of the plantar side of the foot using a modified “PRC” mask (Novel GmbH, Munich, Germany) Feature extraction: Overlapping sliding window approach of a single variable over a specific time duration	Gait analysis: Classifying sitting, standing and walking
**Milovic et al. [43]**	2022	Single-point film pressure sensors: Textile pressure sensor made of a low-density polyethylene (LDPE) sheet (ANT006BCB), integrated in expandable pants	Classification: **Random forest (RF),** time series forest (TSF), and multi-representation sequence learner (Mr-SEQL)	Classification accuracy: **RF: 91.22%** TSF: 90.53% Mr-SEQL: 78.97%	Data pre-processing: (1) Outlier elimination; (2) Fifth-order low-pass Butterworth filter Feature extraction: TS Fresh Relevant-Feature Extractor function	Gait analysis: Detection of gait phases
**Mutlu et al. [14]**	2007	Single-point film pressure sensors: Force-sensitive resistor pressure sensors (FSR, Interlink Electronics, Inc., Shenzhen, China)	Classification: **Simple logistic**, naïve bayes (NB), artificial neural network (multi-layer perceptron) and support vector machine (SVM)	Classification accuracy: **Simple logistic: 82.5%** NB: 74.9% Multi layer perception: 79.1% SVM: 78.5%	Feature extraction: (1) Position and size of the bounding box; (2) Distance between the bounding boxes; (3) Distance and angle between the centers of the pressures; (4) Centers, radii and orientations of two ellipses fit to the pressure areas	Sitting ergonomics: Classification of sitting postures/activities
**Rihar et al. [48]**	2014	Film pressure sensor matrix: 2 CONFORMat systems, Model 5330, (Tekscan Inc., Boston, MA, USA) and 6 IMUs (STMicroelectronics, Plan-les-Ouates, Swiss)	Image recognition		-	Health care: Observation of newborn children
**Rodríguez et al. [71]**	2020	Film pressure sensor matrix: Pressure mat sensor (BodiTrak BT3510, Vista Medical GmbH, Vienna, Austria)	Classification: Convolutional neural network (CNN)	Classification accuracy: Model A: 98.8% Model B: 99.0%	Data pre-processing Fuzzy representation	Health care: Monitoring of elderly patients
**Roh et al. [16]**	2018	Load cells: 4 low-cost load cells (P0236-I42, Hanjin Data Corp., Gimpo, Republic of Korea)	Classification: **Support vector machine (SVM)**, linear discriminant analysis (LDA), quadratic discriminant analysis (QDA), naïve bayes (NB), and random forest (RF), decision tree (DT)	Classification accuracy: **SVMrbf: 97.20%** SVM_lin_: 86.27% LDA: 88.56% QDA: 89.56% NB: 84.33% RF: 93.17% DT: 76.79%	Feature extraction: Sensor fusion	Sitting ergonomics: Sitting postures/activities
**Sazonov et al. [51]**	2011	Single-point film pressure sensors: Five force-sensitive resistors (FSRs) (Interlink Electronics, Inc., Shenzhen, China) and a 3D accelerometer (LIS3L02AS4)	Classification: Support vector machine (SVM)	Classification accuracy: On full sensor set: 95.2% optimized sensor set: 98%	No feature extraction	Gait analysis: Gait activities
Sazonov et al. * [52]	2015	Single-point film pressure sensors: “SmartShoe” device: 5 force-sensitive resistors (FSR) (Interlink Electronics, Inc., Shenzhen, China) and a 3D accelerometer (ADXL335)	Classification: **Support vector machine (SVM)**, multinomial logistic discrimination (MLD), multilayer perceptron (MLP)	Classification Error: Manual activity annotation: 5.0% **SVM: 5.0%** MLD: 5.5% MLP: 5.1%	Feature extraction: Mean value (mean), entropy (ent) and standard deviation (std).	Gait analysis: Gait activities
**Seo et al. [72]**	2021	Film pressure sensor matrix	Classification: Convolutional neural network (CNN)	Classification accuracy: CNN: 99.84%	-	Sitting ergonomics: Sitting postures
**Tam et al. [102]**	2019	Single-point film pressure sensors: 8 pressure sensors (FlexiForce A301, Tekscan Inc., Boston, MA, USA) MU (MPU-6050, InvenSense, San Jose, CA, USA)	Regression: Deep neural network (DNN)	Regression accuracy: MSE: >0.00021 R^2^: <0.93	Feature extraction: 224 features using 3 convolution layers	Gait analysis: Gait activities
Tang et al. * [107]	2012	Single-point film pressure sensors: 2 “SmartShoe” devices and 5 force-sensitive resistors (FSRs) (Interlink Electronics, Inc., Shenzhen, China) and a 3D accelerometer	Classification: Support vector machine (SVM)		No feature extraction	Gait analysis: Gait activities
**Tang et al. [108]**	2014	Single-point film pressure sensors: 2 “SmartShoe” devices, 5 force-sensitive resistors (FSRs) (Interlink Electronics, Inc., Shenzhen, China) and a 3D accelerometer	Classification: Support vector machine (SVM), multilayer perceptron (MLP)	Classification accuracy: SVM: 97.0% SVM_rej_: 98.7% MLP: 97.3% MLP_rej_: 99.8%	Feature extraction: Mean value, minimum value, standard deviation, entropy, variance, maximum value, number of mean crossings (NMC), mean absolute deviation (MAD) and the ratio between the root mean square of wavelet coefficients	Gait analysis: Gait activities
**Tessendorf et al. [109]**	2009	Film pressure sensor matrix: Conformat (Tekscan Inc., Boston, MA, USA)	Classification: Unsupervised learning with database of data prototypes	Classification accuracy: 91%	-	Sitting ergonomics: Sitting postures
**Tsutsui et al. [97]**	2023	Single-point film pressure sensors: 2 FSR-402-short sensors(Interlink Electronics, Inc., Shenzhen, China)	Analytic classification	Classification accuracy: 75%	-	Gait analysis: Gait activities
**Wu et al. [94]**	2022	Single-point film pressure sensors: Flexible pressure measurement units	Classification	Classification accuracy: 4 regions: 80% 8 regions: 84% FCM model: 95%	Data pre-processing: Fuzzy C-Means Clustering Feature extraction: 4 regions 8 regions FCM model	Sports science: Detection of gait phases in ice and snow sports
**Yin et al. [59]**	2021	Single-point film pressure sensors: Two FSR pressure sensors (LOSON LSH-10)	Classification: Simulated-annealing-algorithm-based support vector machine (SA-SVM), support vector machine (SVM)	Classification accuracy: SA-SVM: 94.8% ± 0.75% SVM: 89.24% ± 2.17%	Data pre-processing: Butterworth low-pass filter (cut-off frequency: 10 Hz) Feature extraction: mRMR algorithm	Gait analysis: Locomotion pattern recognition
**Yousefi et al. [64]**	2011	Film pressure sensor matrix	Classification: K-nearest neighbor (kNN)	Classification accuracy: kNN-ICA: 94.3% kNN-PCA: 97.7%	Data pre-processing: Filtering and histogram equalization	Health care: Patient monitoring
Zemp et al. * [60]	2016	Single-point film pressure sensors: FSR 406, (43.69 mm square sensor, thickness: 0.45 mm; Interlink Electronics, Inc., Shenzhen, China)	Classification: Support vector machine (SVM), multinomial regression (MNR), boosting, neural network (NN), **random forest (RF)**, and a combination of boosting	Classification accuracy: SVM: 82.7% MNR: 87.8% Boosting: 90.4% NN: 90.4% **RF: 90.9%** Combination: boosting NN, RF: 90.8%	Feature extraction: Median of a one-second duration	Sitting ergonomics: Classification of sitting postures
**Zhang et al. [65]**	2022	Single-point film pressure sensors: Flexiforce sensor A301 (Tekscan, Inc., Boston, MA, USA) MEMS Sensor MPU9250	Classification: **Established extreme learning machine (ELM),** K-nearest neighbor (kNN)	Classification accuracy: **ELM (energy feature): 97.4%** ELM (energy entropy feature): 96.6% kNN (energy feature): 95.4% kNN (energy entropy feature): 95.0%	Feature extraction: **(1) Wavelet packet energy features ** (2) Wavelet packet energy entropy features	Gait analysis: Gait pattern recognition
**Zhao et al. [34]**	2021	Film pressure sensor matrix: 2 XSensor pressure mats (Model PX100:48.48.02, XSensor, Calgary, AB, Canada)	Classification: **Random forest (RF)** and deep learning (DL)	Classification accuracy: RF (F1): 52.2% **RF (F2): 80.5%** DL: 42.9%	Feature extraction: F1: absolute sensor values **F2: sensor values relative to the beginning of the task**	Automotive design: Driver’s seat position design
Zhao et al. * [74]	2021	Film pressure sensor matrix: 2 Xsensor pressure mats (Model PX100:48.48.02, (XSensor, Calgary, AB, Canada)	Classification: **Random forest (RF)** support vector machine (SVM), multilayer perceptron (MLP), naïve bayes (NB), and k-nearest neighbor (kNN)	Classification accuracy: **RF: 86%** NB: 76% SVM: 70% MLP: 74% k-NN: 78%	Feature extraction: Body pressure distribution (BPD)-based feature extraction	

**Table 6:** Summary of relevant studies for DHM-based human posture estimation and the analysis of the used input data sources, digital human model, optimization criterium, and application scenarios (* marks the extra added reports, after the structured literature review).

Author	Year	Input Data Source	Digital Human Model	Optimization Criterium	Application Scenario
**Barman et al. [82]**	2022	Virtual force vectors: Load of a box	Dynamic human model: 2D three-compartment controller fatigue model, 10 DOF	Optimization of joint fatigue	Industrial ergonomics: Lifting task
**Choi et al. [76]**	2020	Load cell: 6-axis force–torque sensor (nano25, ATI, Apex, NC, USA)	Dynamic human model: 3D human model	Optimization of joint torques	Sports science: Estimating swing movements in golfing
**Clever et al. [93]**	2018	Virtual pressure matrix: Virtual smart sensor mattress	Human body shape model		Sleep research: Estimation of human sleeping posture
**Davoudabadi et al. [79]**	2015	Virtual force vectors: Load of a dumbbell	Musculoskeletal human model: Anybody modelling system (AnyBody Technology A/S, Aalorg, Denmark)	Minimization of muscle effort	Sports science: Snatch weightlifting
Davoudabadi et al. * [110]	2016	Virtual force vectors: Load to the human hand–arm system	Musculoskeletal human model: Anybody modelling system (AnyBody Technology A/S, Aalorg, Denmark)	Minimization of muscle effort	Sports science: Using a sailing winch
**Howard et al. [98]**	2011	Film pressure sensor matrix	Dynamic human model 3D human model	Optimization of joint torques	Sitting ergonomics: Optimization of wheel chair design
**Kwon et al. [85]**	2017	Virtual force vectors: Personal body load	Dynamic human model: Generator of body (GEBOD) and SantosTM (SantosHuman Inc., Coralville, IA, USA)	Minimization of discomfort function and energy cost	Gait analysis: Estimation of joint angle and gait movements
**Liu et al. [84]**	2009	Virtual force vectors: External load on hands	Dynamic human model: Software SNOPT and SantosTM (SantosHuman Inc., Coralville, IA, US)	Optimization of joint torques	Product design: Posture estimation with dynamic human model for external loads
**Lu et al. [89]**	2011	Virtual force vectors	Human body shape model: University of Michigan Three-Dimensional Static Strength Prediction Program (3DSSPP)		Industrial ergonomics
**Mao et al. [99]**	2021	Virtual pressure matrix	Human body shape model: User avatar (self-designed)		Sitting ergonomics: Estimation of different sitting postures on different chair designs
**Marler et al. [77]**	2011	Virtual force vectors: Load of a box	Dynamic human model: Software SNOPT and SantosTM (SantosHuman Inc., Coralville, IA, USA)	Minimization of maximum joint torques	Industrial ergonomics: Analysis of box lifting tasks
**Nakajima et al. [80]**	2022	Virtual force vectors: Contact forces for handling a box	Musculoskeletal human model: Self-designed musculoskeletal hand model	Minimization of muscle effort	Industrial ergonomics: Evaluation of product design and for manual tasks
**Potash et al. [86]**	2022	Virtual force vectors:	Human body shape model: JACK (Siemens Industry Software Inc., Plano, TX, USA)		Industrial ergonomics: Work space optimization
**Rahmati et al. [78]**	2014	Virtual force vectors: Load of a dumbbell	Dynamic human model: 3D human model	Optimization of joint torques	Sport science: Snatch weightlifting techniques
**Ramdani et al. [81]**	2006	Virtual force vectors	Dynamic human model: Four-bar linkage human model	Hypothesis of quasi-static equilibrium	Health care
**Rothaug et al. [88]**	2000	Virtual force vectors	Human body shape model: RAMSIS (Human Solutions GmbH, Kaiserslautern, Germany)		Automotive design
**Salahi et al. [111]**	2016	Virtual force vectors	Dynamic human model: 3D human model	Optimization of joint torques	Fundamental ergonomics research
**Seitz et al. [87]**	2005	Virtual force vectors	Human body shape model: RAMSIS (Human Solutions GmbH, Kaiserslautern, Germany)		Automotive design
**Van Geffen et al. [100]**	2009	Load cells: Two multi-axis load cells (ATI mini 45, ATI Industrial Automation, NYC, USA)	Dynamic human model 3D human model		Sitting ergonomics: Estimation of different sitting postures on different chair design
Wirsching et al. * [90]	2012	Virtual force vectors: Interaction force on hand–arm system	Human body shape model: RAMSIS (Human Solutions GmbH, Kaiserslautern, Germany)		Automotive design: Evaluation of distances in cockpit design
**Yang et al. [42]**	2006	Virtual force vectors: Contact forces for handling objects	Dynamic human model: Self-designed dynamic hand model	Minimization of joint displacement	Fundamental ergonomics research: Dynamic model for hand posture estimation
**Zhang et al. [37]**	2021	Load cells	Dynamic human model		Sports science: Riding a bike
**Zou et al. [101]**	2011	Virtual force vectors: Human body mass	Dynamic human model		Sitting ergonomics: Estimation of different sitting postures

**Table 7:** Summary and qualitative ranking of the main estimation methods and main input data sources for force-based human posture estimation identified in the structured literature review.

Estimation Methods	ML-Based Methods				DHM-Based Methods			
	Image Recognition	Classification		Regression	Dynamic Human Model	Human Body Shape Model		Musculo-Skeletal Human Model
Input Data Sources	Load Cells		Single-Point Film Pressure Sensors		Film Pressure Sensor Matrix		Virtual Force Vectors	
Required expert knowledge for using the methods	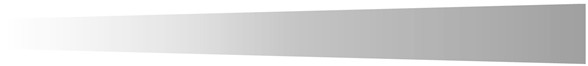							
Necessary knowledge about the application	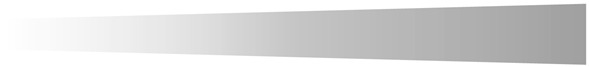							
Effort for adaptation to a new application	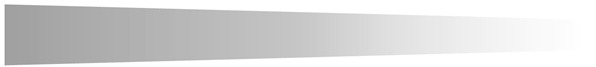							
Detail level of output posture	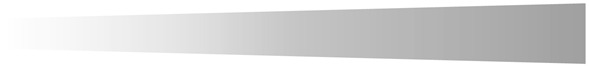							
Potential for automatization	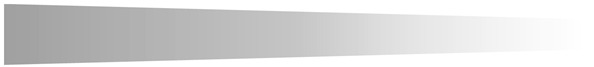							
Potential for sensor integration	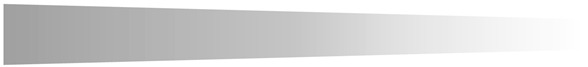							

The ranking criteria are the required expert knowledge for using the methods, the necessary knowledge about the application context and the effort for adaptation to a new application as requirements for the utilization of the methods. Furthermore, the detail level of the output posture, the potential for automatization and the potential for sensor integration are the ranking criteria that describe the advantages of the methods and sensors in an application. The potential for automatization is describing the standardization of the application process—comparable to a manufactory process—and is significant for a mass application of smart products. The bar graphs illustrate the intensity (light = low, dark = high) for each criterion. The methods and sources most often used in the studies are underlined.

## Data Availability

All data generated are included in this study.
